# The impact of enterprise digital transformation on employees’ intrinsic motivation: the mediating role of justice perceptions

**DOI:** 10.3389/fpsyg.2025.1544103

**Published:** 2025-12-08

**Authors:** Xiaorui Chang, Junqing Yang

**Affiliations:** 1 School of Economics and Management, Taiyuan University of Science and Technology, Taiyuan, China; 2 School of Business Administration, Shanxi University of Finance and Economics, Taiyuan, China

**Keywords:** enterprise digital transformation, employee justice perceptions, means-ends fusion theory, information processing, employee intrinsic motivation

## Abstract

Enterprise digital transformation is an important means for enterprises to seek innovation and development, and intrinsic motivation serves as the foundation for employees’ innovation, a crucial indicator of a successful enterprise digital transformation is the enhancement of employees’ intrinsic motivation. However, there are relatively few existing studies focusing on the positive relationship between enterprise digital transformation and employees’ intrinsic motivation, so it is necessary to further explore the mechanism through which enterprise digital transformation stimulates employees’ intrinsic motivation. Based on the structural intrinsic motivation perspective, data from 607 in-service employees were collected using a three-wave approach. The research results, derived from path analysis and moderating effect analysis methods, indicate that enterprise digital transformation has an motivational effect on employees’ intrinsic motivation, and employees’ distributive justice, procedural justice, and interactional justice perceptions all play significant mediating roles, while information processing job characteristics show significantly different moderating effects on different motivational paths: When information processing are low, the distributive justice path loses its motivational effect, while the motivational effect of the interactional justice path significantly decreases when information processing are high, and the moderating effect on the procedural justice path is always insignificant. These findings highlight the micro-positive effects of enterprise digital transformation, provide new explanations for the existing research disagreement, and provide theoretical and practical insights for implementing rational, orderly, and targeted digital transformation strategies for employees with different job characteristics in practice.

## 1 Introduction

Enterprise digital transformation has gradually become an important means to increase productivity and drive high-quality innovation in enterprises (Cheng et al., 2023; Wu et al., 2024), and by reshaping business processes and work methods through digital technology (Plekhanov et al., 2023), enterprise digital transformation has had a significant impact on both enterprises and employees (Kensbock and Ckmann, 2020). Existing literature has focused on the role of enterprise digital transformation in promoting innovation in firms (Chen and Kim, 2023; He et al., 2024; Li et al., 2022; Peng and Tao, 2022; Zhao et al., 2022), alongside the positive effects on employees’ creativity and innovation (Jia et al., 2024; Lanzolla et al., 2020). Simultaneously, intrinsic motivation is a major prerequisite for employees to achieve innovative results (Hennessey and Amabile, 1998; Zhang et al., 2022); however, existing literature mostly believes that enterprise digital transformation may reduce employees’ autonomy at work (Manroop et al., 2024; Xie et al., 2021) and thus has a negative impact on their intrinsic motivation (Arnaud and Chandon, 2013; Ller et al., 2018; Ollier-Malaterre et al., 2019). Therefore, past studies on the positive role of enterprise digital transformation are contradictory at the level of employees’ behavior and motivation. The divergence of existing literature not only fails to provide psychological motivation support for the macro-positive results of enterprise digital transformation, but also may hinder employees from forming a positive perception of it. This, in turn, is not conducive to developing the digital economy. It is thus necessary to explore in detail how enterprise digital transformation can positively influence employees’ intrinsic motivation.

The negative view of existing literature that enterprise digital transformation is detrimental to employees’ intrinsic motivation may be related to its predominantly adopted theoretical perspective, the self-determination theory (Kensbock and Ckmann, 2020; Xie et al., 2021). Self-determination theory is the dominant theory in research on intrinsic motivation in organizational contexts (Deci et al., 2017; Gagné and Deci, 2005; Gerhart and Fang, 2015; Kruglanski et al., 2018), which argues that managerial practices that convey approving or controlling messages to employees have an impact on intrinsic motivation, and that when management practices are not conducive to meeting employees’ needs for autonomy, competence, or relatedness at work, this will reduce their intrinsic motivation (Deci et al., 2017). The digital transformation of enterprises retrieves and stores employees’ work information and monitors their work behavior (Kensbock and Ckmann, 2020; Manroop et al., 2024), thus adversely affecting employees’ intrinsic motivation by undermining their work autonomy. Existing studies have ignored the fact that enterprise digital transformation is a management practice with the double-edged characteristics of “Taylorism” and “Autonomy” (Dabić et al., 2023; Kirchner et al., 2023; Parker and Grote, 2022). Exploring how employees cognitively integrate and evaluate its different pros and cons may provide new mechanistic insights and theoretical contributions to existing research (Bankins et al., 2024). Past work has focused too much on the impact of digital transformation on employees’ need for autonomy at work (Kirchner et al., 2023), ignoring the potential mediating mechanisms of other important intrinsic goals of employees, such as the goal of justice (Bankins et al., 2024), and the moderating effect of job characteristics through their impact on employees’ cognitive tendencies (Morandini et al., 2023; Verplanken and Orbell, 2022).

Based on the means-ends fusion theory, this study investigated whether the activity of enterprise digital transformation can be associated and fused with employees’ justice perceptions and thus affect the mechanism of their intrinsic motivation by constructing a model of moderated mediators, and examined the moderated boundary role of information processing. Based on this, this study addresses the academic debates sparked by digital “Taylorism” and “Autonomy” (Dabić et al., 2023; Kirchner et al., 2023; Parker and Grote, 2022; Bankins et al., 2024) and advances the research on the relationship between enterprise digital transformation and employees’ intrinsic motivation.

## 2 Theory and hypotheses

### 2.1 Means-ends fusion theory: a structural intrinsic motivation theory

Means-ends fusion theory is a theory that focuses on the associative fusion between external activities and individuals’ internal goals, and how this fusion influences intrinsic motivation. It holds that positive associations between external activities and internal goals will lead to positive changes in intrinsic motivation (Fishbach and Woolley, 2022). Unlike self-determination theory, which focuses on how the satisfaction of certain basic personal needs affects intrinsic motivation, means-ends fusion theory emphasizes the importance of the fusion between activities and internal goals: when individuals experience external activities as the realization of their internal goals, their intrinsic motivation will be enhanced (Kruglanski et al., 2018). Means-ends fusion theory deconstructs intrinsic motivation as the degree to which external activities help individuals achieve their internal goals, and is also known as the structural intrinsic motivation theory (Kruglanski et al., 2018; Phillips and Mullan, 2023).

Means-ends fusion theory points out that the influence of external activities on internal goals has three characteristics: first, the associative fusion between activities and goals is unidirectional—goals can affect the perception of activities, but the activities themselves do not affect the characteristics of goals; second, goals must be in an activated state; third, the more important personal goals are when fused with activities, the stronger individuals’ intrinsic motivation will be (Fishbach and Woolley, 2022). The means-ends fusion theory is a structural theory of intrinsic motivation that is gradually gaining attention (Grant et al., 2011; Phillips and Mullan, 2023), providing a new theoretical perspective for studying how digital transformation affects employees’ intrinsic motivation.

### 2.2 Enterprise digital transformation and employees’ intrinsic motivation

Enterprise digital transformation refers to the use of digital technologies by enterprises to reshape their existing business models and workflows (Plekhanov et al., 2023), which often leads to the digitization of employees’ workflows and task content (Ullrich et al., 2023), and thus changes the employees’ work context. Changes in the work context can trigger subjective cognitive interpretations and evaluations, affecting employees’ intrinsic motivation to work and creating incentivizing or disincentivizing effects (Barrick et al., 2012). Some scholars have referred to the way employees work under the digital transformation of enterprises as “algorithmic management” (Dabić et al., 2023). Algorithmic management includes both negative factors such as limiting work autonomy (Newlands, 2021) and positive factors such as improving work efficiency through information and algorithms (Kellogg et al., 2020). According to the means-ends fusion theory, the presence of an extrinsic activity with characteristics of opposite potency can trigger the fusion of enterprise digital transformation with employees’ intrinsic goals and enhance intrinsic motivation if there is a personal intrinsic goal that is similar to the characteristics of the activity. Research in cognitive neuroscience has also found that in a new work situation, employees prioritize the activation of their own goal-oriented system, call on the cognitive maps accumulated in their work experience, and judge the level of effort they need to put in the new situation, as well as the value of reaching the goal (O’Doherty et al., 2017). By calculating the sum of the expected costs and benefits of taking positive action (Hosokawa et al., 2013), individuals will establish a causal link between the activity and the goal (Liljeholm et al., 2011). Thus, how employees perceive their overall personal benefits under the digital transformation of enterprises is an important process mechanism to explain how their intrinsic motivation changes.

### 2.3 Mediating role of justice perceptions

In an organizational context, justice perceptions refers to the employees’ perception of the reasonableness of the procedures, processes, and outcomes of what they give and what they receive in terms of compensation from the organization by comparing themselves and others; it is the primary goal of the employee when working in an organization (Colquitt and Zipay, 2015). Perceptions of injustice reduces employees’ intrinsic motivation and work effort (Leete, 2000). Justice includes three dimensions, namely distributive justice, procedural justice, and interactional justice (Kim and Leung, 2007). Distributive justice refers to the perceived fairness of receiving compensation relative to what is paid for at work, procedural justice refers to the perceived consistency and lack of bias in the way and process by which compensation is provided by the organization, and interactional justice refers to the perceived respect and explanations from supervisors when compensation decisions are made (Colquitt, 2001).

The salient feature of enterprise digital transformation is the increasing character of work in enterprise information, computing, communication, and connectivity (Barišić et al., 2021; Huang et al., 2021; Vial, 2019); the main challenge for employees is the increase in digital information diversity and information density (Ller et al., 2018). Employees need to invest more cognitive resources to cope with it, and by anticipating the overall balance between their own payoffs and rewards, employees can judge whether digital transformation is meaningful to them or not (Ullrich et al., 2023). Thus, enterprise digital transformation triggers employees to weigh the fairness of costs and payoffs, which is uniquely related to their goal of justice; according to the means-ends fusion theory, the two will produce a fusion phenomenon, which will in turn affect employees’ intrinsic motivation.

Regarding distributive justice, enterprise digital transformation leads to the networking of task processes, digitization of task interfaces, and automation of data exchange and management (Kurek, 2021; Verhoef et al., 2021), which tend to have a positive impact on employees’ task efficiency (Barišić et al., 2021). With the emergence of high-end digital transformation activities such as Big Data and Artificial Intelligence, the potential of enterprise digital transformation to improve employees’ task efficiency is increasing (Barišić et al., 2021; Dabić et al., 2023). In addition, digital work styles reduce the frequency of disruptions at work, allowing employees to be more focused and productive (Golden and Gajendran, 2019); increased employee efficiency represents a reduction in the amount of work time it takes to complete the same task (Parker and Grote, 2022). Employees thus have more flexibility to adjust their contribution to work time, increase time for skill acquisition and training, and even have the opportunity to autonomously achieve work-life balance (Ollier-Malaterre et al., 2019), thus achieving a balance between payoffs and rewards and increasing their perception of distributive justice. Accordingly, this study proposed the following hypothesis:

*H1a*: Enterprise digital transformation positively influences employees’ distributive justice perceptions.

Regarding procedural justice, in an enterprise digital transformation scenario, employees’ work status and results can be tracked and announced online in real time through digital performance monitoring and other technologies (Li, 2020). The evaluation of employees’ work quality is more objective and comparable, and the compensation provided by the organization to employees will be based on their objective work performance and no longer on the number of hours worked (Horváth and Szabó, 2019). The performance evaluation and compensation transparency provided by the digital transformation of the organization will urge the management of the organization to improve the consistency and unbiasedness of the compensation decision-making process, and employees’ perception of procedural justice will be enhanced as a result. Accordingly, this study proposed the following hypothesis:

*H1b*: Enterprise digital transformation has a positive impact on employees’ procedural justice perceptions.

With respect to interactional justice, the digital transformation of enterprises has led to the networking and platformization of work communication, and the communication and collaboration among employees and between employees and supervisors are increasingly dependent on virtualized online tools (Ller et al., 2018). Enterprise digital transformation has triggered the reduction of humanized social interactions in the work environment, and it is difficult to form special emotional ties between supervisors and employees (Xie et al., 2021). The information exchange between supervisors and employees tends to be programmed, which reduces the opportunities for supervisors to use their power for personal gain, and the reward and punishment decisions for employees are characterized by dehumanization (Cascio and Montealegre, 2016). The difficulty of establishing a unique social relationship between employees and supervisors leads to a tendency for supervisors to treat employees with equal social respect and disclosure of information, which will enhance employees’ interactional justice perceptions. Accordingly, this study proposed the following hypothesis:

*H1c*: Enterprise digital transformation has a positive impact on employees’ interactional justice perceptions.

Enterprise digital transformation is an activity that transforms work contexts (Kensbock and Ckmann, 2020); its motivational attributes are mainly derived from task characteristics or social characteristics that allow individuals to experience their hard work guided by their own goals as meaningful (Barrick et al., 2012), leading to intrinsic motivation (Fishbach and Woolley, 2022). According to the means-ends fusion theory, since enterprise digital transformation is uniquely associated and fused with employees’ distributive justice perceptions, procedural justice, and interactional justice, employees will view enterprise digital transformation as realizing their goals of the three dimensions of justice, which enhances the perceived significance of enterprise digital transformation, thus enhancing employees’ intrinsic motivation.

What may raise suspicion are the possible privacy breaches, behavioral surveillance, and other stressful features of digital tasks (Cascio and Montealegre, 2016). Do they also fuse with employees’ justice perceptions, which in turn affects intrinsic motivation? Research in cognitive neuroscience suggests that the direction and intensity of an individual’s regulation of their own cognition and behavior in task situations that combine both motivational and pressures can affect intrinsic motivation (Botvinick and Braver, 2015); the antecedents that support individuals to actively self-regulate in stressful situations are no longer dominated by resource theories (Botvinick and Braver, 2015). Moreover, reward mechanisms are considered to be the main source of positive self-regulatory and behavioral control (Botvinick and Braver, 2015; O’Doherty et al., 2017); individuals will integrate and convert positive and negative incentives in the environment into a common currency, judge the overall benefits of their own cognitive and behavioral regulation, and then influence their intrinsic motivation and decision-making (O’Doherty et al., 2017). It can be seen that even if there are some situational pressure characteristics of digital transformation in enterprises, employees can also achieve fusion with their justice perceptions by judging the overall benefits. Meanwhile, the literature on employee motivation suggests that monitoring employees can reduce the phenomenon of employees receiving unfair rewards through “invisible” work withdrawal (Gerhart and Fang, 2015). Enterprise digital transformation will thus significantly enhance employees’ overall interests and increase management transparency and effectiveness (Demerouti, 2022). Its overall positive impact on employees’ perceptions of distributive, procedural, and interactional justice will outweigh the negative impact, thereby helping to enhance employees’ intrinsic motivation. Accordingly, this study proposed the following hypothesis:

*H2*: Employees’ distributive, procedural and interactional justice perceptions play a mediating role in the positive impact of enterprise digital transformation on employees’ intrinsic motivation.

### 2.4 The moderating role of information processing

According to the means-ends fusion theory, any variable that affects the degree of association between activities and goals affects the fusion of enterprise digital transformation and the justice perceptions, which in turn affects the intrinsic motivation of employees (Fishbach and Woolley, 2022), thus showing a moderating effect. The impact of enterprise digital transformation on employees’ cognition and motivation is induced by changing the way work is done, especially as it alters the informational and extended nature of traditional work tasks (Kozanoglu and Abedin, 2020; Lanzolla et al., 2020), placing high demands on employees’ ability to integrate and utilize information (Verhoef et al., 2021; Vial, 2019). Employees need to shift their perceptions and strive for synergy with new technologies to enhance their ability to utilize intelligent algorithmic information and immediate task information to increase their work efficiency (Kozanoglu and Abedin, 2020). Therefore, the level of employees’ information processing ability is an important factor that affects the degree of fusion of enterprise digital transformation and employees’ justice perceptions. What are the sources of differences in employees’ information processing ability? Cognitive ability, characterized by educational qualifications, is usually considered to be the source of differences; however, more and more highly educated employees are now deployed in low-skilled positions, triggering overqualification and negative work attitudes (Erdogan and Bauer, 2021), thus suggesting that differences in job characteristics may influence employees’ information processing abilities more than differences in educational qualifications.

Research in cognitive neuroscience has also provided evidence for the moderating effects of job characteristics by suggesting that an individual’s ability to process a task corresponds to a stimulus–response association, or learned habit, which is defined as a direct context-response association learned through repeated rewards (Phillips and Mullan, 2023), derived from repeated contextual cues and corresponding behaviors in stable environments. This significantly affects an individual’s cognitive reaction time to cope with novel situations, leading to individual differences in cognition and tolerance (Verplanken and Orbell, 2022). In the organization context, employees’ work habits are mainly derived from their own job work characteristics rather than their own educational characteristics (Parker and Grote, 2022); therefore, differences in employees’ positional job characteristics are an important factor influencing the fusion of digital transformation and their justice perceptions in the enterprise. Existing studies have shown that information processing requirements have a significant impact on information processing capabilities (Wang, 2003). Therefore, this study argues that among different types of job characteristics, information processing is an important factor influencing the differences in information processing capabilities among employees. Based on the means-ends fusion theory, information processing exerts a moderating effect.

Information processing refer to the extent to which employees need to attend to and process data or other information on the job, reflecting the high level of monitoring and active information acquisition that characterizes the work of the position (Morgeson and Humphrey, 2006). Employees in positions characterized by high information processing are better at dealing with the information processing and integration challenges posed by enterprise digital transformation, which frees up more learning and attentional cognitive resources that enhance the ability to satisfy their intrinsic goals (Parker, 2014). The fusion of enterprise digital transformation with employees’ justice perceptions and its impact on intrinsic motivation can have differential effects depending on the information processing of employees’ positions (Kirchner et al., 2023).

Regarding the path of distributive justice gain, employees with high information processing are better at dealing with the information processing and integration challenges posed by enterprise digital transformation, thus freeing up more learning and attentional cognitive resources (Parker, 2014). They increase their speed of adaptation to digital tasks, improve work efficiency and save work time faster, and gain more time to pursue personal goals and other resources, which enhances their perception of the uniqueness and timeliness associated with enterprise digital transformation and perceptions of distributive justice. Furthermore, enterprise digital transformation places higher demands on the load and bandwidth of employees’ processing of work information, and employees with high information processing are also more accustomed to cognitive processing of work information (Phillips and Mullan, 2023). The high degree of similarity and repetitive coupling between the two increases employees’ perceptions of distributive justice; according to the means-ends fusion theory, the above characteristics of information processing will increase the degree of perceived fusion between enterprise digital transformation and employees’ perception of distributive justice, further leading to their increased intrinsic motivation. Accordingly, this study proposed the following hypothesis:

*H3a*: Information processing positively moderates the effect of enterprise digital transformation on employees’ distributive justice perceptions.

*H3b*: Information processing positively moderates the indirect effect of enterprise digital transformation on employees’ intrinsic motivation through distributive justice.

Regarding the path of procedural justice gain, digital work styles allow employees to receive instant compensation information when completing tasks (Xie et al., 2021). Employees with high information processing have greater information sensitivity and analytical efficiency (Morgeson and Humphrey, 2006), and may more quickly and deeply perceive the open and transparent characteristics of the compensation decision-making process and outcomes brought about by the enterprise digital transformation. This enhances their perception of the uniqueness and timeliness of the association between enterprise digital transformation and the perception of procedural justice, which in turn enhances their intrinsic motivation more significantly. Accordingly, this study proposed the following hypothesis:

*H3c*: Information processing positively moderates the effect of enterprise digital transformation on employees’ procedural justice perceptions.

*H3d*: Information processing positively moderates the indirect effect of enterprise digital transformation on employees’ intrinsic motivation through procedural justice.

In contrast to the above moderating mechanisms, in terms of the interactional justice gain path, enterprise digital transformation has changed the traditional way in which information resources were mainly held and delivered by supervisors (Lepine et al., 2016), making employees more dependent on the enterprise digital platform for information. Employees with high information processing are more accustomed to actively searching and processing information (Morgeson and Humphrey, 2006), and have a greater ability to analyze information about the process and outcomes of compensation; therefore, they have a lower need for interactional justice from supervisors who provide information about social respect and decision-making (Ller et al., 2018). The perceived convergence of enterprise digital transformation with employees’ perception of interactional justice (obtained from supervisors) will be weakened by high information processing; consequently, the ability of enterprise digital transformation to indirectly enhance employees’ intrinsic motivation through the sense of interactional justice will be diminished. Accordingly, this study proposed the following hypothesis:

*H3e*: Information processing negatively moderates the effect of enterprise digital transformation on employees’ interactional justice perceptions.

*H3f*: Information processing negatively moderates the effect of enterprise digital transformation on employees’ interactional justice perceptions.

**Figure 1:**
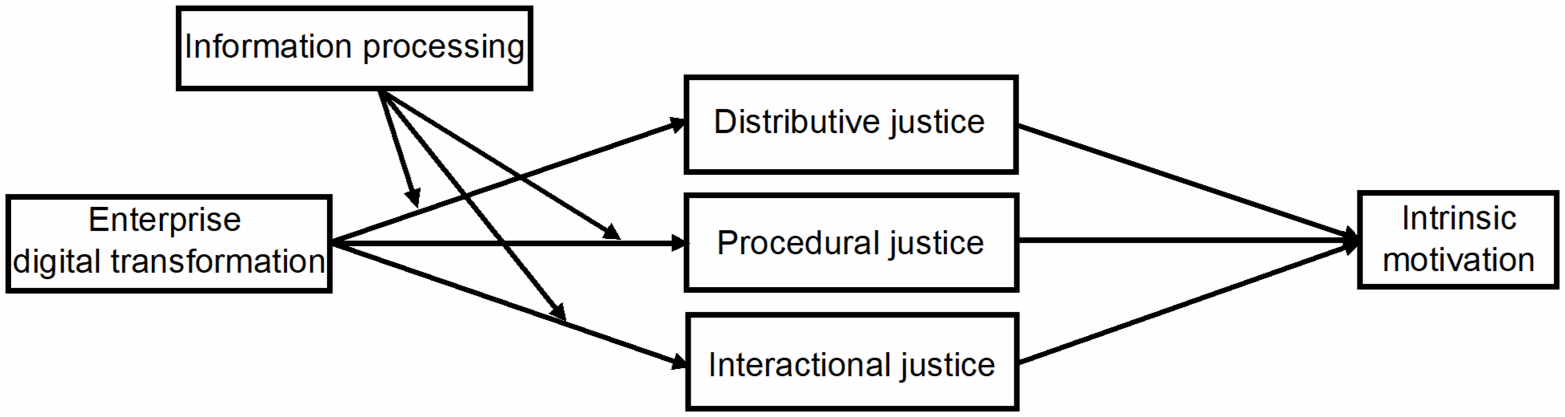
Theoretical model.

In summary, the theoretical model proposed in this study is shown in Figure 1.

## 3 Methods

### 3.1 Participants

Data were collected from full-time employees recruited through the Credamo platform. While the sample was obtained through self-selection (Berndt, 2020; Galloway, 2005), due to the lack of a reference population sampling frame (Keiding and Louis, 2018), self-selection bias may have potential impacts on sample representativeness and exaggeration or misleading of research results, it reflects a diverse group of full-time employees, allowing for meaningful analysis of the research hypotheses. *Credamo* is an authoritative online workforce research platform for academic research in China, similar to Amazon’s *Mechanical Turk (MTurk)* platform in the U.S. The *Credamo* survey platform sends survey invitations to registered full-time corporate employees, individuals with a credit score below 70 points are excluded during the invitation phase, while samples with high credit scores are eligible to apply for survey participation. Before the test, the researchers informed the participants of the study’s purpose in writing, emphasized that the survey was conducted anonymously and that participants’ information would be strictly protected, and the participants could only continue to take part in the test after providing informed consent. The survey respondents are full-time employees working in major Chinese cities such as Beijing and Shanghai, and across various industries including finance and manufacturing. In terms of geography and industry, the sample is representative of characteristics under the Chinese cultural context. To minimize common methodological bias, the research was divided into three phases with multiple follow-ups, and 2 RMB was given to the employees who participated at the end of each research phase as payment. High-quality participants were screened on the research platform before the start of the research by controlling conditions such as credit score and the number of times they answered questions; attention screening questions were set to eliminate participants who did not fill out the questionnaires seriously online, to improve the research quality.

Questionnaires were collected from 800 full-time employees at time point 1, and participants were required to report their demographic information, enterprise digital transformation, and information processing variables. The 2nd questionnaire collection was conducted after an interval of 2 weeks, and the questionnaires were distributed only to the 800 participants who participated in the 1st questionnaire collection. They were required to report their perceptions of distributive, procedural, and interactional justice variables, and 650 questionnaires were recovered, with a return rate of 81%. The 3rd questionnaire collection was started again after an interval of 2 weeks, and the questionnaires were distributed only to the 650 participants who participated in the 2nd questionnaire collection; they were required to report on personal compensation, job complexity job characteristics and intrinsic motivation variables. Finally, 607 valid questionnaire scores were obtained, with a valid recovery rate of 93.4%. Since there are no significant differences in demographic characteristics and other attributes between the attrition samples and the samples finally included in the analysis, this study concludes that the attrition of initial samples will not cause bias in the empirical results (Walters, 2021). Among the 607 participants, there were 237 males (39.0%), 370 females (61.0%), and 567 with a Bachelor’s degree or above (93.4%). Table 1 summarizes the sample’s demographic characteristics.

**Table 1:** Demographic information of the sample.

Variable		Frequency	Percentage (%)
Gender	Male	237	39.0
	Female	370	61.0
Age (years)	21–30	296	48.8
	31–40	256	42.2
	41–50	37	6.1
	51–60	16	2.6
	60 and over	2	0.3
Education	High school and below	10	1.7
	specialized training school	30	4.9
	Bachelor	420	69.2
	Master	142	23.4
	PhD and above	5	0.8
Tenure	Within 1 year	68	11.2
	1–3 years	152	25.0
	3–5 years	154	25.4
	More than 5 years	233	38.4
Job type	Front-line positions	82	13.5
	R&D positions	224	36.9
	Management positions	301	49.6

*N* = 607.

### 3.2 Measures

To ensure measurement validity, the questionnaires in this study were based on well-established scales that have been used many times in authoritative journals, and all the scales were measured using a seven-point Likert scale ranging from “Strongly Disagree” to “Strongly Agree.”

Enterprise digital transformation (abbreviated as EDT): A five-item scale developed by HuQing was used (Hu, 2020), with representative questions such as “Our enterprise comprehensively promotes digital design, manufacturing, and management.”

Information processing (abbreviated as IP): A four-item scale developed by Morgeson and Humphrey was used (Morgeson and Humphrey, 2006), with representative questions such as “The job requires me to monitor a great deal of information.”

Distributive justice (abbreviated as DJ): A three-item scale developed by Kim was used (Kim, 2004, as cited in Kim and Leung, 2007), with representative questions such as “The rewards I received here are quite fair.”

Procedural justice (abbreviated as PJ): A three-item scale developed by Kim was used (Kim, 2004, as cited in Kim and Leung, 2007), with representative questions such as “This organization makes decisions in fair ways.”

Interactional justice (abbreviated as IJ): A three-item scale developed by Kim was used (Kim, 2004, as cited in Kim and Leung, 2007), with representative questions such as “My supervisor treats me fairly.”

Intrinsic motivation (abbreviated as IM): A four-item scale developed by Grant was used (Grant, 2008), with representative questions such as “Because I find the work engaging.”

Control variables: First, this study controlled for common demographic variables such as gender, age, education, tenure, and job type of the participants. Second, compensation (abbreviated as CP) is an important antecedent that affects employees’ justice perceptions, which was controlled in this study and measured using a seven-item item scale developed by Chuang and Liao (2010), with representative questions such as “On average the pay level (including incentives) of our employees is higher than that of our competitors.”

### 3.3 Data analysis

SPSS 26.0 software was used to analyze the measurement validity and reliability of the questionnaire, descriptive statistical information of variable means, variances and correlation coefficients, Harmer’s one-way test of variance, variable covariance and hierarchical regression analyses, and to analyze and test the direct, mediating, and moderating effects between variables and their 95% confidence intervals using the PROCESS program and bootstrap method. The Mplus 8.3 software was used to test the discriminant validity of the variables and the common method bias of the latent factors.

## 4 Results

### 4.1 Reliability test and validated factor analysis

The reliability indicators of the variables measured by the scale were better than the recommended values [factor loadings > 0.5, composite reliability (CR) values > 0.7, average variance extracted (AVE) values > 0.5] (see Table 2). The variables were tested for discriminant validity by confirmatory factor analysis (CFA) using Mplus 8.3, in addition to the hypothetical seven-factor model (six variables of the main model and one control variable). Six competing models were set up to compare the goodness-of-fit based on the conceptual characteristics of the variables, and the results showed that the hypothetical model fit indicators of the seven-factor model were all better than the recommended values, χ^2^ = 724.949, degree of freedom (*df*) = 356, *χ*^2^/*df* = 2.036, comparative fit index (CFI) = 0.954, Tucker-Lewis index (TLI) = 0.948, standardized root mean squared residual (SRMR) = 0.036, root mean square error of approximation (RMSEA) = 0.041. The fitting effect was significantly better than the other competing models, which showed that the hypothetical model had a high distinguishing validity (see Table 3).

**Table 2:** Results of the reliability of variable measurement.

Variable	Factor loading	Cronbach’s *α*	CR	AVE	$\sqrt{\mathrm{AVE}}$	VIF
EDT	0.717–0.759	0.784	0.854	0.540	0.735	1.803
IP	0.787–0.857	0.829	0.887	0.662	0.814	1.093
DJ	0.814–0.856	0.783	0.875	0.699	0.836	2.121
PJ	0.772–0.827	0.727	0.847	0.649	0.806	1.865
IJ	0.782–0.834	0.740	0.853	0.659	0.812	1.514
IM	0.860–0.916	0.912	0.938	0.791	0.889	reference
CP	0.699–0.761	0.853	0.889	0.534	0.731	2.513

*N* = 607. EDT, enterprise digital transformation; IP, information processing; DJ, distributive justice; PJ, procedural justice; IJ, interactional justice; IM, intrinsic motivation; CP, compensation.

**Table 3:** Confirmatory factor analysis results.

Model	*χ* ^2^	*df*	*χ*^2^/*df*	CFI	TLI	SRMR	RMSEA
7-factor model	724.949	356	2.036	0.954	0.948	0.036	0.041
EDT, IP, DJ, PJ, IJ, CP, IM							
6-factor model	846.688	362	2.339	0.940	0.932	0.039	0.047
DJ + PJ, IJ, EDT, IP, CP, IM							
5-factor model	1103.162	367	3.006	0.908	0.899	0.049	0.057
DJ + PJ + IJ, EDT, IP, CP, IM							
4-factor model	1919.014	371	5.173	0.807	0.789	0.074	0.083
IP + DJ + PJ + IJ, EDT, CP, IM							
3-factor model	2084.995	374	5.575	0.787	0.769	0.077	0.087
EDT + IP + DJ + PJ + IJ, CP, IM							
2-factor model	2232.717	376	5.938	0.769	0.751	0.079	0.090
EDT + IP + DJ + PJ + IJ + CP, IM							
1-factor model	2899.057	377	7.690	0.686	0.662	0.085	0.105
EDT + IP + DJ + PJ + IJ + CP + IM							

*N* = 607. EDT, enterprise digital transformation; IP, information processing; DJ, distributive justice; PJ, procedural justice; IJ, interactional justice; IM, intrinsic motivation; CP, compensation.

### 4.2 Control and testing of common method deviations

To minimize the common method bias, before beginning the research, the methods of subject qualification screening, anonymous responses, attention test, and the collection of questionnaires in three waves with an interval of 2 weeks were controlled. To increase the rigor of the study, common method latent factor method (ULMC) was used to test common method bias, and after adding common method variance factors to the seven-factor model, RMSEA = 0.041, CFI = 0.965, TLI = 0.956, and SRMR = 0.029, which compared to the theoretical model ∆RMSEA = 0, ∆CFI = 0.011, ∆TLI = 0.008, and ∆SRMR = −0.007; none of these values exceeded the limit of 0.02. Using the Harmer one-way test for common method bias, the unrotated variance extraction of the first principal component explained 35.737% of the total variation, which did not exceed the limit of 40%, suggesting that the common method bias in this study is not serious (Podsakoff et al., 2003). In addition, multicollinearity was analyzed, and the average variance inflation factor (VIF) value for each variable was 1.818, with a maximum value of 2.513 (see Table 2 for details), which is much less than the limit of 10. This indicated that there is no severe covariance between the variables and regression analysis can be performed (Shrestha, 2020).

### 4.3 Descriptive statistics

The means, standard deviations, and correlation coefficients of the variables are shown in Table 4 below. The enterprise digital transformation was significantly and positively correlated with employees’ perceptions of distributive, procedural, and interactional justice (*r* = 0.497/0.530/0.525, respectively, *p* < 0.01), and employees’ perceptions of distributive, procedural, and interactional justice were significantly and positively correlated with their intrinsic motivation (*r* = 0.614/0.537/ 0.435, respectively, *p* < 0.01). The relationship between the variables was in line with the theoretical hypotheses expected.

**Table 4:** Descriptive statistical analysis.

Variable	Mean	SD	1	2	3	4	5	6	7	8	9	10	11	12
Gender	0.610	0.488	1											
Age	1.640	0.743	−0.029	1										
Education	3.170	0.595	0.078	−0.041	1									
Tenure	2.910	1.037	−0.015	0.644**	0.035	1								
Job type	2.230	0.692	0.083*	0.059	0.201**	0.082*	1							
EDT	3.525	0.487	−0.160**	0.192**	0.070	0.278**	0.084*	1						
IP	5.457	0.943	−0.109**	0.091*	0.048	0.023	0.154**	0.242**	1					
DJ	5.677	0.899	−0.095*	0.162**	0.064	0.281**	0.071	0.497**	0.103*	1				
PJ	6.002	0.706	−0.059	0.183**	0.036	0.262**	0.041	0.530**	0.172**	0.547**	1			
IJ	5.889	0.803	−0.085*	0.151**	0.021	0.244**	0.056	0.525**	0.248**	0.389**	0.447**	1		
IM	5.596	1.189	0.015	0.260**	0.082*	0.372**	0.059	0.527**	0.159**	0.614**	0.537**	0.435**	1	
CP	5.207	0.550	−0.028	0.190**	0.100*	0.298**	0.147**	0.551**	0.175**	0.705**	0.624**	0.442**	0.589**	1

*N* = 607. *p < 0.05, **p < 0.01 (two-tailed). EDT, enterprise digital transformation; IP, information processing; DJ, distributive justice; PJ, procedural justice; IJ, interactional justice; IM, intrinsic motivation; CP, compensation.

### 4.4 Hypothesis testing

The results of the hierarchical regression of the variables are shown in Table 5. After the control variables were included in the model, the enterprise digital transformation had a significant positive effect on the employees’ distributive justice perceptions (M2, *β* = 0.135, *p* < 0.01), procedural justice (M6, *β* = 0.259, *p* < 0.01), and interactional justice (M10, *β* = 0.391, *p* < 0.01), and the hypotheses H1a, H1b, and H1c were supported. The mediation effect reliability was tested using the Bootstrap method with repeated sampling 10,000 times using the PROCESS program Model 4 (see Table 6), and the indirect effect value of enterprise digital transformation affecting intrinsic motivation through the distributive justice perceptions was 0.045, with a 95% CI = (0.011, 0.087). The indirect effect affecting intrinsic motivation through the procedural justice perceptions was 0.049, 95% CI = (0.016, 0.096), and the indirect effect value of influencing intrinsic motivation through the perceptions of interactional justice was 0.047, 95% CI = (0.009, 0.093); thus, the mediating effect was significant in all cases. Hypothesis H2 was supported.

**Table 5:** Hierarchical regression results for direct, mediation, and moderating effects.

Variable	DJ				PJ			
	M1	M2	M3	M4	M5	M6	M7	M8
Gender	−0.073*	−0.054	−0.057*	−0.055	−0.035	0.003	0.006	0.009
Age	−0.032	−0.035	−0.030	−0.030	0.023	0.017	0.013	0.013
Education	0.003	−0.001	0.000	0.002	−0.014	−0.020	−0.021	−0.018
Tenure	0.100*	0.085*	0.079*	0.083*	0.071	0.043	0.048	0.052
Job type	−0.031	−0.031	−0.026	−0.021	−0.050	−0.051	−0.056	−0.051
CP	0.684**	0.616**	0.618**	0.608**	0.606**	0.475**	0.473**	0.462**
EDT		0.135**	0.143**	0.166**		0.259**	0.252**	0.276**
IP			−0.042	−0.051			0.036	0.026
EDT × IP				0.067*				0.073*
*R* ^2^	0.510	0.522	0.524	0.528	0.401	0.445	0.446	0.451
Δ*R*^2^	0.510**	0.012**	0.002	0.004*	0.401**	0.044**	0.001	0.005*
F	104.216**	93.596**	82.279**	74.226**	66.91**	68.658**	60.258**	54.517**

Variable	IJ				IM				
	M9	M10	M11	M12	M13	M14	M15	M16	M17
Gender	−0.070	−0.013	−0.002	−0.006	0.035	0.075*	0.093**	0.092**	0.093**
Age	−0.014	−0.022	−0.038	−0.039	0.042	0.036	0.048	0.045	0.047
Education	−0.018	−0.029	−0.031	−0.035	0.031	0.024	0.024	0.027	0.029
Tenure	0.132**	0.089	0.107*	0.101*	0.190**	0.160**	0.131**	0.127**	0.120*
Job type	−0.004	−0.006	−0.024	−0.031	−0.046	−0.048	−0.037	−0.030	−0.031
CP	0.406**	0.208**	0.200**	0.215**	0.529**	0.390**	0.184**	0.128**	0.118*
EDT		0.391**	0.365**	0.331**		0.276**	0.230**	0.195**	0.164**
DJ							0.335**	0.312**	0.309**
PJ								0.148**	0.133**
IJ									0.090*
IP			0.131*	0.145**					
EDT × IP				−0.104**					
*R* ^2^	0.215	0.316	0.331	0.341	0.394	0.444	0.498	0.510	0.515
Δ*R*^2^	0.215**	0.101**	0.015**	0.010**	0.394**	0.050**	0.053**	0.012**	0.005*
F	27.336**	39.444**	36.972**	34.275**	65.007**	68.378**	74.045**	68.905**	63.264**

*N* = 607. *p < 0.05, **p < 0.01 (two-tailed). EDT, enterprise digital transformation; IP, information processing; DJ, distributive justice; PJ, procedural justice; IJ, interactional justice; IM, intrinsic motivation; CP, compensation.

**Table 6:** Results of the reliability test for the mediating effect.

Mediator variable	Estimate	S.E.	Boot 95% CI	
			Lower limit	Upper limit
DJ	0.045	0.019	0.011	0.087
PJ	0.049	0.021	0.016	0.096
IJ	0.047	0.021	0.009	0.093

*N* = 607. DJ, distributive justice; PJ, procedural justice; IJ, interactional justice.

**Figure 2:**
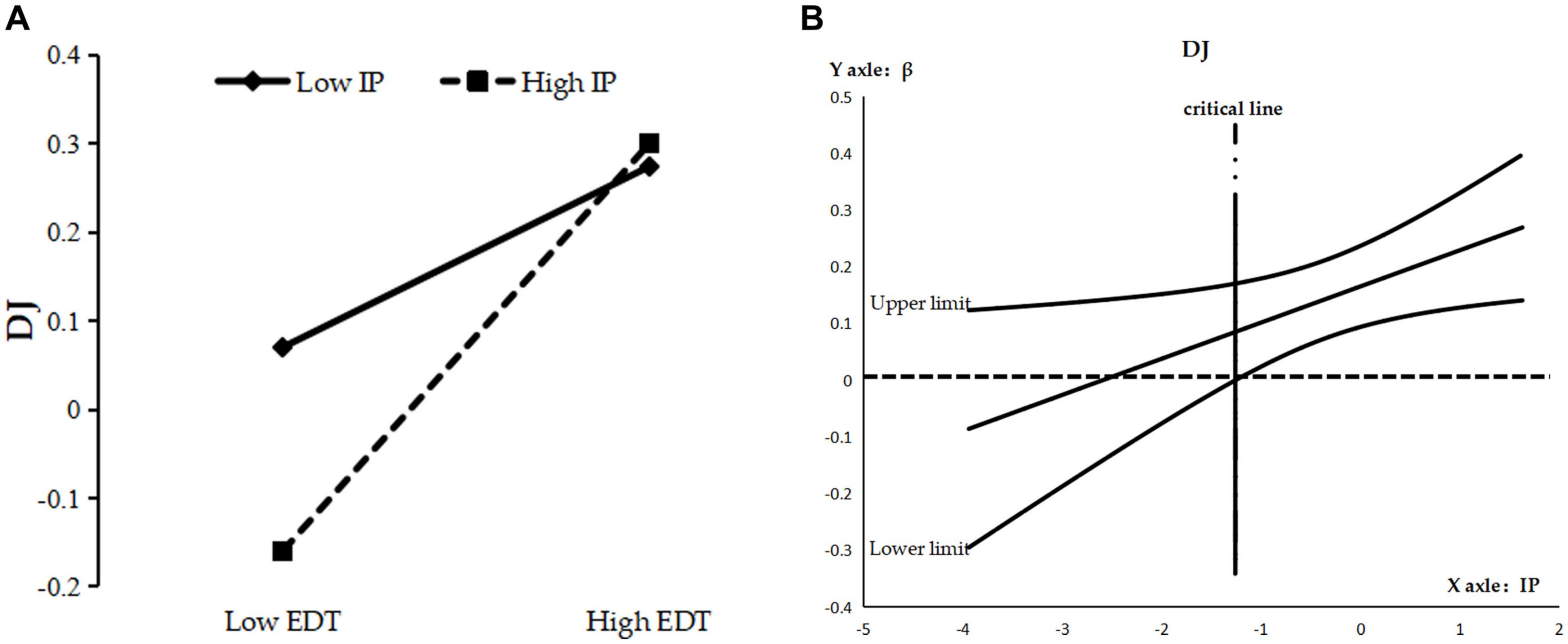
**(A)** Simple slope diagram for DJ. **(B)** J-N diagram for DJ.

**Figure 3:**
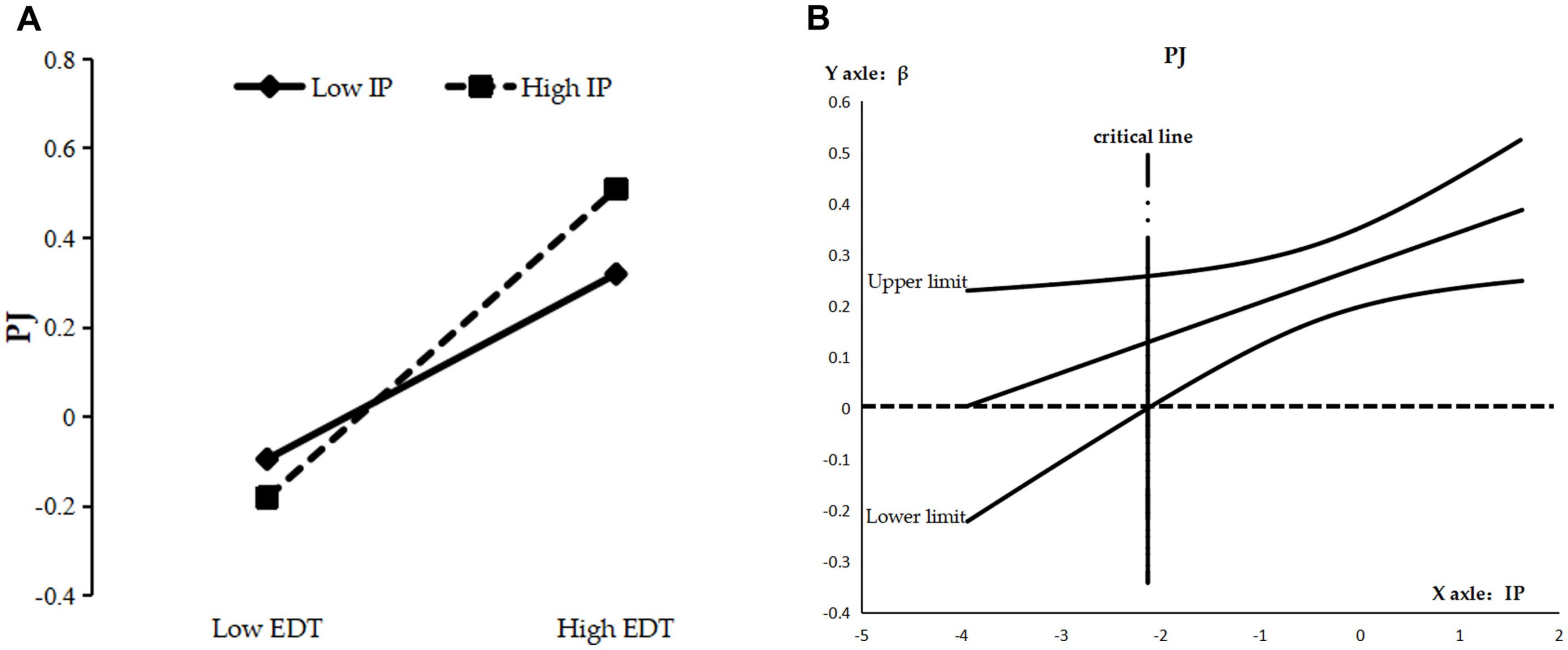
**(A)** Simple slope diagram for PJ. **(B)** J-N diagram for PJ.

**Figure 4:**
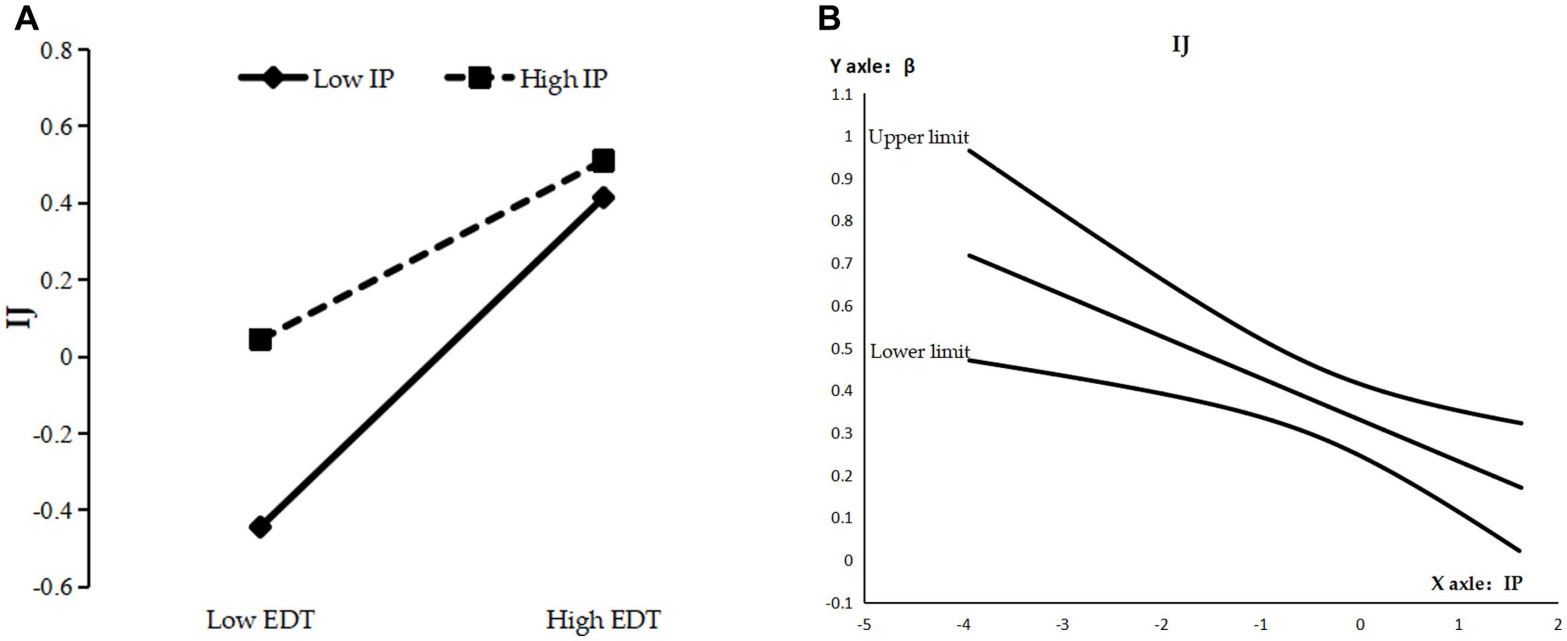
**(A)** Simple slope diagram for IJ. **(B)** J-N diagram for IJ.

As can be seen from Table 5, the interaction term of enterprise digital transformation and information processing significantly and positively affected distributive (M4, *β* = 0.067, *p* < 0.05), procedural (M8, *β* = 0.073, *p* < 0.05), and interactional justice (M12, *β* = −0.104, *p* < 0.01), and the direct moderating effect was initially established. The reliability of the direct moderating effect was tested by the Bootstrap method using Process program Model 1 for repeated sampling 10,000 times. By plotting a simple slope diagram (Figures 2A, 3A, 4A), it can be seen that when the information processing were 1 standard deviation (SD) above the mean, the positive effect of enterprise digital transformation on distributive justice (*β* = 0.229, SE = 0.052, *p* < 0.01) (Figure 2A) and procedural justice (*β* = 0.345, SE = 0.056, *p* < 0.01) (Figure 3A) was enhanced, while that on the perceptions of interactional justice (*β* = 0.232, SE = 0.061, *p* < 0.01) was relatively weakened (Figure 4A). By plotting the J-N (Johnson–Neyman) plot of moderating effects with 95% confidence interval boundaries (Figures 2B, 3B, 4B), it can be seen that the moderating effects on distributive justice and procedural justice were significant and increasing when the information processing were greater than −1.261 SD (Figure 2B) and −2.130 SD (Figure 3B), respectively, while their moderating effects on interactional justice were always decreasing constantly and significantly (Figure 4B). Hypotheses H3a, H3c and H3e were supported.

The indirect moderating effect of information processing and its reliability were tested by Bootstrap method using PROCESS program Model 7 with 10,000 repetitive samples, and the results are shown in Table 7. For the mediating path of distributive justice, when information processing were high (M + 1SD), the value of the indirect effect was significantly higher at 0.077 [95% CI = (0.030, 0.141)], whereas the indirect effect was not significant [95% CI = (−0.010, 0.074)] when information processing were low (M-1SD), with a significant difference in the indirect effect value of 0.043 [95% CI = (0.007, 0.111)], Hypothesis H3b was supported. For the mediating path of procedural justice, contrary to the hypotheses, the high (M + 1SD) and low (M − 1SD) difference in the indirect moderating effect of information processing was not significant [95% CI = (−0.032, 0.060)]; in summary, Hypothesis H3d was not supported.

**Table 7:** Results of the indirect moderating effect test.

Path	IP	Estimate	S.E.	Boot 95% CI
EDT → DJ → IM	M − 1SD	0.034	0.021	(−0.010, 0.074)
	M + 1SD	0.077	0.029	(0.030, 0.141)
	Difference (Δ)	0.043	0.027	(0.007, 0.111)
EDT → PJ → IM	M − 1SD	0.039	0.022	(0.010, 0.094)
	M + 1SD	0.065	0.024	(0.021, 0.113)
	Difference (Δ)	0.026	0.023	(−0.032, 0.060)
EDT → IJ → IM	M − 1SD	0.052	0.023	(0.011, 0.102)
	M + 1SD	0.028	0.016	(0.004, 0.064)
	Difference (Δ)	−0.024	0.015	(−0.059, −0.001)

*N* = 607. EDT, enterprise digital transformation; IP, information processing; DJ, distributive justice; PJ, procedural justice; IJ, interactional justice; IM, intrinsic motivation.

For the mediating effect of interactional justice, the indirect effect value was significantly higher at 0.052 [95% CI = (0.011, 0.102)] when the information processing was low (M-1SD), and the difference in the indirect effect for the high (M + 1SD) and low (M − 1SD) information processing was significant lower at −0.024 [95% CI = (−0.059, −0.001)]; hypothesis H3f was supported.

### 4.5 Robustness analysis

Information processing may not be the only characteristics that affect employees’ information processing ability and have a moderating effect; this study further analyzed whether job complexity characteristics (abbreviated as JCC) had a moderating effect. Job complexity characteristics refer to the complexity and difficulty of performing the tasks of an employee’s position. Contrary to the simplicity of the tasks, job complexity requires more skills and mental demands from employees, which affects their skill learning and stress coping habits at the workplace (Morgeson and Humphrey, 2006). It may also affect employees’ data and information processing skills, which in turn affect the degree of perceived fusion between enterprise digital transformation and employees’ perceptions of justice, with direct and indirect moderating effects on the hypothesized model. Job complexity characteristics were measured using a six-item scale developed by Morgeson and Humphrey (2006). In this study, the Cronbach’s alpha coefficient was 0.911, the factor loadings were 0.877 to 0.905, the CR value was 0.938, and the AVE value was 0.790. The reliability and validity of the variable were high.

Model 1 and Model 7 of the PROCESS program were used with the 10,000 times Bootstrap method to test the direct and indirect moderating effect, and the results are shown in Table 8 below. Job complexity characteristics had a significant direct positive moderating effect on distributive and procedural justice, and a significant direct negative moderating effect on interactional justice; the 95% CI values excluded 0, consistent with the direct moderating effect of information processing. However, the indirect moderating effect of job complexity was quite different from the model hypothesis, and its indirect moderating effect difference on the mediating effect of distributive justice was 0.044, with 95% CI = (−0.002, 0.099); on the mediating effect of procedural justice was 0.024, with 95% CI = (−0.018, 0.061); and on the mediating effect of interactional justice was −0.021, with 95% CI = (−0.026, 0.001); the CIs all contained 0. Thus, the indirect moderating effect of job complexity characteristics was not significant. It can be seen that job complexity characteristics cannot replace information processing to produce a significant indirect moderating effect.

**Table 8:** Results of the direct and indirect moderating effect test.

Path	JCC	Estimate (direct)	S.E.	Boot 95% CI	Estimate (indirect)	S.E.	Boot 95% CI
EDT → DJ → IM	M − 1SD	0.099	0.039	(0.022, 0.176)	0.033	0.019	(−0.005, 0.073)
	M + 1SD	0.231	0.049	(0.134, 0.327)	0.077	0.028	(0.026, 0.135)
	Difference (Δ)				0.044	0.025	(−0.002, 0.099)
EDT → PJ → IM	M − 1SD	0.219	0.043	(0.136, 0.303)	0.041	0.021	(0.011, 0.092)
	M + 1SD	0.346	0.053	(0.241, 0.450)	0.065	0.024	(0.024, 0.115)
	Difference (Δ)				0.024	0.020	(−0.018, 0.061)
EDT → IJ → IM	M − 1SD	0.449	0.047	(0.357, 0.542)	0.054	0.024	(0.011, 0.104)
	M + 1SD	0.275	0.059	(0.158, 0.391)	0.033	0.018	(0.005, 0.076)
	Difference (Δ)				−0.021	0.007	(−0.026, 0.001)

*N* = 607. EDT, enterprise digital transformation; JCC, job complexity characteristics; DJ, distributive justice; PJ, procedural justice; IJ, interactional justice; IM, intrinsic motivation.

## 5 Discussion

### 5.1 Conclusion

From the perspective of structural intrinsic motivation, this study constructed a mechanism model of enterprise digital transformation positively affecting employees’ intrinsic motivation through the fusion of employees’ goals regarding distributive, procedural, and interactional justice. Compared with existing studies that focus on the controversy between “Taylorism” and “Autonomy” triggered by enterprise digital transformation, this study further reveals the important role of perceived justice in influencing employees’ intrinsic motivation (Dabić et al., 2023; Kirchner et al., 2023; Parker and Grote, 2022). The information processing significantly moderate the positive fusion of enterprise digital transformation with the distributive justice and procedural justice, and generate a significant direct positive moderating effect, alongside a significant indirect positive moderating effect on the gain path of distributive justice, this finding is consistent with previous studies that identify procedural justice as a key factor in employee satisfaction(Simmers and Mcmurray, 2018; Olafsen et al., 2015). On the other hand, consistent with the hypothesis, the information processing had a significant direct and indirect negative moderating effect on the gain path of interactional justice. Overall, the research findings support the positive influence process of enterprise digital transformation on employees’ intrinsic motivation, thereby achieving the main objectives of the study.

### 5.2 Theoretical contributions

First, this study provides an explanatory mechanism for when enterprise digital transformation motivates employees’ intrinsic motivation. Existing studies argue that digital transformation poses challenges to human resource management (Dabić et al., 2023), highlighting its negative impact on employees’ intrinsic motivation (Arnaud and Chandon, 2013; Ller et al., 2018; Manroop et al., 2024; Ollier-Malaterre et al., 2019; Xie et al., 2021). Compared with existing studies, this study are more supportive of the positive effects of enterprise digital transformation on employees’ intrinsic motivation. The mediating role of employees’ perceptions of justice goals reveals how employees cognitively integrate the positive and negative features of enterprise digital transformation. The findings contribute to understanding the positive micro-psychological mechanisms behind enterprise digital transformation leading to employees’ intrinsic motivation.

Second, this study advances the theoretical and empirical application of the structural intrinsic motivation theory in organizational contexts. Structural intrinsic motivation theory, represented by the means-ends fusion theory, not only considers the importance of intrinsic goal activation but also expands the sources of intrinsic motivation from the perspective of goal diversification (Fishbach and Woolley, 2022; Phillips and Mullan, 2023). However, structural intrinsic motivation theory has been applied mainly in the fields of kinesiology, education, and psychology since its formulation, and research in organizational contexts has yet to advance (Woolley and Fishbach, 2023). This study makes an attempt to explore the application of structural intrinsic motivation theory in organizations; through theoretical and empirical analyses, it shows that structural intrinsic motivation theory can explain the mechanisms and job characteristic boundaries of how enterprise digital transformation activities affect employees’ intrinsic motivation.

Again, the boundary of the moderating mechanisms of enterprise digital transformation affecting employees’ motivation is extended. Existing studies have mainly examined the moderating mechanisms of personal traits and digital activity characteristics (Gagné and Deci, 2005; Liu et al., 2023), ignoring the important moderating effects that job characteristics may have (Maula et al., 2023). Information processing, as a major factor in the formation of employees’ information processing ability, can have a significant effect on the degree of perceived fusion of enterprise digital transformation activities associated with employees’ perception of distributive and interactional justice, moderating the process by which enterprise digital transformation affects employees’ intrinsic motivation (Fishbach and Woolley, 2022). Thus, the empirical analysis extends the moderating boundaries of enterprise digital transformation activities affecting employees’ motivation.

Finally, this study enriches the research on how enterprise digital transformation affects employees’ perception of justice. Existing research on the digital context and employees’ perception of justice has examined a separate dimension of justice (Bankins et al., 2024), lacking systematic analysis and empirical research on how digital transformation affects the content of each dimension; moreover, only experimental manipulation methods have been used to examine research hypotheses (Alder and Ambrose, 2005a, 2005b; Pei et al., 2021; Xie et al., 2021), which has limited the strength of the causal relationship revealed. By establishing the moderated mediator model and examining the relationship between enterprise digital transformation and employees’ perception of distributive justice, procedural justice, and interactional justice, this study makes up for the shortcomings of existing literatures.

### 5.3 Practical implications

The empirical results of this study found that enterprise digital transformation can help improve employees’ intrinsic motivation by improving their justice perception, but their information processing job characteristics have an important moderating role; thus, enterprises should take into account the organization’s job planning and job characteristics of existing employees’ positions, and adopt digital transformation strategies step by step. Specifically, for positions with low information processing, simpler digital transformation techniques should be adopted, and supervisors should explain and communicate compensation decision information to these employees in a timely manner. For positions with high information processing, more advanced digital technology should be adopted as early as possible to make full use of digital and intelligent work information to enhance the work efficiency and distributive justice perceptions of these employees; these employees should also be provided with more objective information on performance and salary decision-making through digital platforms to improve their interactional justice perceptions, thus enhancing their intrinsic motivation. Finally, enterprise digital transformation significantly improves employees’ perceptions of procedural justice; for enterprises where employees generally have a strong need for information disclosure, they should adopt digital transformation technologies as early as possible.

### 5.4 Limitations and future directions

The study has certain limitations. First, the data in this study were all from questionnaires, and the scales were filled in by employees recruited online; although the techniques of multi-stage research, eligibility, and attention judgment were used to control the quality of the questionnaire responses, the problem of homologous bias could not be completely avoided, and future research should conduct more detailed variable measurements and studies through field research and empirical sampling methods. Second, in the empirical analysis of this study, besides demographic variables, only the impact of employees’ compensation variables (which mainly measures salary and benefits) on their perception of justice was additionally controlled. In fact, mixed elements such as development opportunities, recognition, and work-life balance all influence employees’ justice perception (Milkovich et al., 2017). Future studies should investigate and control more types of compensation to enhance the reliability of the model. Future research may consider controlling for more components of compensation and verifying whether the conclusions of the model still hold. Finally, the sample of this study cannot represent research conclusions in Western cultural contexts, future research should examine Western contexts to assess cross-cultural validity.

## Data Availability

The raw data supporting the conclusions of this article will be made available by the authors, without undue reservation.
