# Supplementary material for: The impact of enterprise digital transformation on employees’ intrinsic motivation: the mediating role of justice perceptions
Source: Front Psychol. 2025 Dec 8;16:1544103. doi: 10.3389/fpsyg.2025.1544103 (PMC12722889; doi:10.3389/fpsyg.2025.1544103)
Supplement: Supplementary file 1 [file Supplementary_file_1.docx]

**Appendix A**

1. Enterprise digital transformation (Hu, 2020)

Our enterprise adopts digital technologies to transform and upgrade existing products, services, and processes.

Our enterprise fully promotes digital design, manufacturing, and management.

Our enterprise develops digital products and services.

Our enterprise is willing to invest efforts in vigorously promoting and popularizing digital skills and management knowledge.

Our enterprise reaches a consistent internal view that adopting digital technologies and digital management is conducive to enterprise development.

2. Information processing (Morgeson and Humphrey, 2006)

The job requires me to monitor a great deal of information.

The job requires that I engage in a large amount of thinking.

The job requires me to keep track of more than one thing at a time.

The job requires me to analyze a lot of information.

3. Distributive justice (Kim 2004, as cited in Kim and Leung, 2007)

The rewards I received here are quite fair.

I believe that I am being rewarded fairly here at work.

I receive fair rewards in this organization.

4. Procedural justice (Kim 2004, as cited in Kim and Leung, 2007)

This organization makes decisions in fair ways.

The procedures used to handle organizational issues are fair.

The rules and procedures to make decisions are fair.

5. Interactional justice (Kim 2004, as cited in Kim and Leung, 2007)

My supervisor treats me fairly.

In interpersonal encounters, my supervisor gives me a fair treatment.

The way my supervisor treats me is fair.

6. Intrinsic motivation (Grant, 2008)

Because I care about benefiting others through my work.

Because I want to help others through my work.

Because I want to have positive impact on others.

Because it is important to me to do good for others through my work.

7. Compensation (Chuang and Liao, 2010)

On average the pay level (including incentives) of our employees is higher than that of our competitors.

Employee salaries and rewards are determined by their performance.

The store rewards employees for new ideas for improving customer services.

The store provides a variety of benefits.

The store does not attach importance to the fairness of compensation/rewards. (R)

Employees receive monetary or nonmonetary rewards for great effort and good performance.

The store gives special rewards to employees who are excellent in serving customers.
